# Is physical fitness associated with brain structure and function in Parkinson’s disease?

**DOI:** 10.1007/s11682-026-01098-x

**Published:** 2026-03-11

**Authors:** Adrian R. Corfitsen, Mikkel K. E. Nygaard, Simon F. Eskildsen, Ulrik Dalgas, Martin Langeskov-Christensen

**Affiliations:** 1 Exercise Biology, Department of Public Health, Aarhus University, Dalgas Avenue 4, 8000 Aarhus C, Denmark; 2 Public Health Aarhus, Aarhus Municipality, Ceres Allé 13, 8000 Aarhus C, Denmark; 3 Center of Functionally Integrative Neuroscience, Department of Clinical Medicine, Aarhus University, Universitetsbyen 3, 8000 Aarhus C, Denmark; 4 Department of Neurology, Viborg Regional Hospital, Heibergs Alle 2, 8800 Viborg, Denmark; 5 Department of Clinical Medicine, Aarhus University, Palle Juul-Jensens Blvd. 82, 8200 Aarhus N, Denmark

**Keywords:** Parkinson’s disease, Exercise therapy, Neurodegeneration, Magnetic resonance imaging

## Abstract

We investigated associations between physical fitness (cardiorespiratory fitness (VO_2_max), peak muscle power (P_max_)) and brain structure (magnetic resonance imaging (MRI) measures) and function (cognition) in people with Parkinson’s disease (pwPD). Simple and multiple linear regression analyses were performed using quantitative susceptibility mapping, diffusion imaging, and volumetric MRI data from 105 pwPD. Simple regression analyses showed significant positive associations between VO_2_max/P_max_ and whole-brain volume (*r*^2^ = 0.11/0.12), the Symbol Digit Modalities Test (SDMT) (*r*^2^ = 0.18/0.21), the Montreal Cognitive Assessment (MoCA) (*r*^2^ = 0.12/0.12) as well as volume of white and gray matter structures (total white/gray matter, putamen, caudate, pallidus, thalamus). When adjusted for age and sex, associations between VO_2_max and cognition (SDMT, MoCA) and between P_max_ and total white matter volume, pallidus volume, and cognition (SDMT) remained significant. Physical fitness was weakly to moderately associated with cognitive function and some MRI markers of neurodegeneration in pwPD. These exploratory findings further support the potential neuroprotective effect of exercise in pwPD and may aid in selecting outcomes in future trials evaluating the neuroprotective effects of exercise.

## Introduction

Parkinson’s disease (PD) is a highly prevalent chronic neurodegenerative disorder characterized by a wide range of motor- and non-motor symptoms (Litvan et al., 2012; Lotharius & Brundin, 2002; MacMahon Copas et al., 2021; Olanow, 1998). Currently, no treatments exist to cure the disease (de Lau & Breteler, 2006), but medical treatments can alleviate symptoms without treating the underlying pathophysiology (Bloem et al., 2021). While searching for curative medical treatments, the identification of non-medical disease-modifying or symptomatic interventions is highly warranted.

Physical activity can be defined as “any bodily movement produced by skeletal muscles that result in energy expenditure”, whereas exercise is typically defined as “a subset of physical activity that is planned, structured, and repetitive and has as a final or an intermediate objective the improvement or maintenance of physical fitness” (Caspersen et al., 1985). Basic physical exercise comprises aerobic exercise (AE) and resistance exercise (RE), which are considered cornerstone exercise modalities (Langeskov-Christensen et al., 2024). Importantly, physical exercise shows promising potential as a feasible and safe disease-modifying treatment for PD (Cugusi et al., 2015; Gamborg et al., 2022; van der Kolk et al., 2019; Schenkman et al., 2018). Moreover, physical exercise is associated with a lower risk of developing PD (Fang et al., 2018), as well as lower all-cause mortality and reduced motor- and non-motor symptoms in people with PD (pwPD) (Jones et al., 2022; Tsukita et al., 2022; Yoon, 2017). Furthermore, previous studies have reported acute exercise-induced increases in brain-derived neurotrophic factor (Hirsch et al., 2018) and increased functional corticostriatal connectivity following 6 months of aerobic exercise in pwPD (Johansson et al., 2022), providing evidence for relevant pathways to a framework for potential explanatory underlying mechanisms. Interestingly, AE is hypothesized to exert neuroprotective effects in PD by reducing oxidative stress and neuroinflammation (Ahlskog, 2018; Landers, 2020), making markers of cardiorespiratory fitness relevant for further investigation (Ferris et al., 2007; Petzinger et al., 2013; Yang et al., 2020). In this regard, the VO_2_max, a performance and health indicator in pwPD (Gamborg et al., 2022), is positively associated with brain volume and cognitive function in numerous non-PD studies (Ahlskog, 2018; Hirsch et al., 2018; Johansson et al., 2022; Yoon, 2017). Taken together, the current evidence supports a potential neuroprotective and disease-modifying effect of AE in pwPD. Oppositely, the effect of exercise on cognitive function in PD has been less intensively investigated although preliminary results show positive effects in studies applying heterogeneous methodologies (Intzandt et al., 2018; Murray et al., 2014; da Silva et al., 2018). At this stage, the neuroprotective effects of RE in PD remain largely unknown, as the majority of the literature has focused on symptomatic effects (Brienesse & Emerson, 2013; Paolucci et al., 2020). Furthermore, a beneficial prophylactic effect of RE on neuroinflammation and cognitive decline has been reported in a PD animal model (Gregório et al., 2022), highlighting the rationale for further exploration of potential neuroprotective effects of RE in PD. Lower extremity peak muscle power (P_max_) is normally improved by RE and is a strong indicator of lower body muscle function, and P_max_ is further associated with both performance and health in pwPD (Gamborg et al., 2022). Thus, more knowledge on associations between P_max_ and neuroprotective parameters is warranted in pwPD.

The main neuropathological characteristic of PD is a loss of dopaminergic neurons in the substantia nigra (SN) and hence dopaminergic innervation to the dorsal striatum (Burciu et al., 2017; Fearnley & Lees, 1991) paralleled by Lewy body aggregation and impaired iron homeostasis in the SN (Langkammer et al., 2012). Histopathology studies show a 30–100% increase in iron concentration in the SN of pwPD compared to healthy controls, which may lead to oxidative stress, inflammation, and neurodegeneration via reactive oxygen species formation, microglial activation, and α-synuclein aggregation (Dusek et al., 2015). Brain iron concentration, particularly in the SN pars compacta (SNpc), is also associated with clinical outcomes, including cognitive decline and Movement Disorder Society-Sponsored Revision of the Unified Parkinson’s Disease Rating Scale (MDS-UPDRS) scores, making it a potential disease progression biomarker (Pyatigorskaya et al., 2020; Thomas et al., 2020). Magnetic resonance imaging (MRI), particularly quantitative susceptibility mapping (QSM), reliably measures iron content in PD (Langkammer et al., 2016; Ravanfar et al., 2021). Free water fraction (FWF), derived from diffusion MRI, is another relevant marker of neurodegeneration, as it reflects extracellular water content and microstructural changes in brain tissue (Burciu et al., 2017). FWF in the posterior part of SN (PSN) has been proposed as a disease progression marker in early-stage PD patients (Burciu et al., 2017). Together, iron concentration and FWF provide complementary insights into PD-related neurodegeneration.

Exercise seems to protect against brain degeneration, while increased iron concentration promotes it. Nonetheless, no study has investigated the associations between VO_2_max (reflecting aerobic fitness), lower extremity peak muscle power (P_max_, reflecting RE training status), and MRI markers of neurodegeneration – including iron concentration, brain morphology, and FWF – in pwPD.

Thus, the primary aim of the present study was to investigate the association between physical fitness (i.e., VO_2_max and P_max_) and MRI markers of neurodegeneration, including iron concentration, brain morphology, and FWF in pwPD. A secondary aim was to examine the relationship between physical fitness and cognitive function. It was hypothesized that physical fitness would be positively associated with cognitive function and negatively associated with MRI markers of neurodegeneration, suggesting a neuroprotective effect.

## Materials & methods

This cross-sectional study used baseline data from a randomized controlled trial (RCT) investigating the potential neuroprotective effects of 24 weeks of supervised moderate to high-intensity AE in 70 low to moderately active pwPD. The RCT was approved by the ethical committee of the Central Denmark Region (record no. 1–10–72–260-18), conducted following the Helsinki Declaration, and registered in the database of the US National Library of Medicine (clinicaltrials.gov; NCT04379778). Additionally, cross-sectional data from 35 highly physically active pwPD were collected. In total, data from 105 pwPD were used in the present study. Of note, another cross-sectional study from the trial examining associations between physical activity levels, VO_2_max, and P_max_ and measures of the MDS-UPDRS and the Parkinson’s disease questionnaire-39 (PDQ-39) has previously been published (Bonde-Jensen et al., 2024).

### Study design and participants

Participants were recruited via the Dept. of Neurology at Aarhus University Hospital, Kolding Hospital, Odense University Hospital, and through local neurological clinics in Esbjerg, Aarhus, Skanderborg, and Horsens. Furthermore, participants were recruited via the Danish PD Society’s website. Potential participants were screened via a phone conversation to check the following eligibility criteria.

To be included, participants had to give their signed informed consent to participate, be older than 40 years, have a clinical diagnosis of idiopathic PD within the past five years, and have a Hoehn & Yahr stage of 3 or lower. They also needed to be able to complete the planned tests and intervention, transport themselves to and from testing and training sessions, and participate in high-intensity aerobic exercise less than twice a week at study entry.

Participants were excluded if they suffered from alcohol abuse, depression, or had a pacemaker. Other exclusion criteria included pregnancy, cardiovascular, respiratory, orthopedic, or metabolic disorders, or other comorbidities that hindered testing or exercise training. Additionally, participants were excluded if they had metallic implants preventing MRI-scanning or a resting blood pressure greater than 160/100.

The categorization of physical activity level into groups of ‘low to moderately’ and ‘highly’ physically active patients was based on self-reported physical behaviour obtained from the phone interview. The interview included specific questions about aerobic exercise habits (i.e., duration, frequency, and intensity of the activities). To determine the intensity of the exercise performed, participants were asked about their sensations of breathing and heart rate during exercise. High-intensity aerobic exercise was defined as a longer-lasting physical activity that caused shortness of breath and/or a high heart rate. Persons with PD who had performed high-intensity aerobic exercise at least twice per week for at least three months were considered highly active, while those who exercised less than two times per week were categorized as low to moderately active.

### Outcomes

#### MRI

MRI measurements were performed by trained personnel using one 3 T MRI scanner (MAGNETOM Skyra; Siemens Medical Systems, Erlangen, Germany). T1-weighted MP2RAGE images were acquired with TR = 5000 ms, TE = 2.98 ms, TI_1_ = 700 ms, TI_2_ = 2500 ms at a spatial resolution of 1 × 1 × 1 mm^3^ and a matrix size of 256 × 256 × 176. Scan time for the MP2RAGE sequence was 11:51 min. QSM of the entire brain was performed using a 3D multi-echo, gradient echo sequence (number of slices = 64, slice thickness = 2 mm, matrix size = 320 × 240, and pixel size = 1.1 mm). Eight echoes from 5 to 47 ms were acquired with TR = 80 ms and flip angle of 15°. Scan time for the QSM was 9:41 min. Phase images were stored for calculating the QSM maps. During scans, participants were placed in a supine position with the head resting in a positioning aid for stabilization purposes, minimizing motion throughout the scan.

For volumetric analysis, T1-weighted images were processed using the framework by Aubert-Broche et al. (2013). First, images went through preprocessing, where noise was suppressed (Coupe et al., 2008; Coupé et al., 2010), intensity inhomogeneity caused by radio frequency coil variations was corrected (Sled et al., 1998), and the brain intensity was scaled to a reference intensity scale. Secondly, each image was registered to an MRI template in MNI (Montreal Neurological Institute) space (ICBM152 nonlinear 9 C) (Aubert-Broche et al., 2013). Lastly, segmentations of the different brain components were done, using a patch-based segmentation method (Coupé et al., 2011; Eskildsen et al., 2012). Volumes of whole-brain (including ventricles and superficial CSF), hippocampus, putamen, caudate, globus pallidus, thalamus, cortex and WM were calculated. Cortex and WM volumes were subdivided into frontal-, occipital-, parietal- and temporal lobes. All volumetric data were normalized by the intracranial volume. For magnetic susceptibility analysis of iron concentration in SN, QSM images were reconstructed using an alteration of the morphology-enabled dipole inversion (MEDI) method as reported by Liu et al. (2011), by substituting Laplacian phase unwrapping with the Speedy rEgion Growing for Unwrapping Estimated phase algorithm (Shmueli, 2019). SN was automatically segmented using atlas-based segmentation by a nonlinear registration (Avants et al., 2014; Xiao et al., 2017) to the multi-contrast Parkinson’s disease template (PD25) followed by transformation of SN atlas from PD25 space to native QSM space.

Diffusion images were acquired with TR = 9300 ms, TE = 101, spatial resolution 2.23 × 2.23 × 2.20 mm, matrix size = 86 × 86 × 60 with the following directions 7 × b = 0 s/mm^2^, 3 × b = 50 s/mm^2^, 6 × b = 200 s/mm^2^, 10 × b = 500 s/mm^2^, 30 × b = 900 s/mm^2^, 16 × b = 1400 s/mm^2^, 30 × b = ^2^500 s/mm^2^. Images were acquired using Anterior–Posterior phase encoding, and an additional 7 images with b = 0 s/mm^2^ were acquired using Posterior-Anterior phase encoding. Scan times for the sequences were 15:11 min and 00:53 min, respectively. FWF was estimated using DiPy 1.7.0 running in Python version 3.11.4, and *b*-values of 2500 s/mm^2^ were excluded in the estimation of FWF. Images were preprocessed by denoising using random matrix theory (Veraart et al., 2016), removal of Gibbs ringing artifacts (Kellner et al., 2016), and correction of motion- and eddy current distortion-induced artifacts (Andersson & Sotiropoulos, 2016). FWF was estimated by using the two-compartment model for diffusion tensor imaging by Henriques et al. (2017). PSN was manually segmented by delineating the region on an average non-linear template of all subjects’ b0 images followed by transforming the segmentation to native space for each subject respectively.

#### VO_2_max

VO_2_max was assessed during a progressive exercise test to exhaustion on a bicycle ergometer (SRM, Jülich, Germany). The test was performed following the protocol previously described by Jensen et al. (2023). Briefly, a five-minute warm-up was performed. Afterward, the load was increased with 10 watts/min (women) or 15 watts/min (men) until exhaustion. This protocol is considered both valid and reliable for pwPD (Jensen et al., 2023).

#### P_max_

Lower extremity muscle peak power was measured during the concentric phase of a chair rise. Peak power was measured using a linear encoder (CHRONOJUMP, Bosco system, v1.8.1, Barcelona, Spain; sampling rate 1000 Hz) and calculated using the built-in software, based on the characteristics of the participants (sex and weight). The participants were instructed to rise from the chair as fast and as powerful as possible, reaching a fully standing erect position without lifting their feet. Two attempts were given, and the highest peak power was used in the analyses.

#### Cognitive function

Cognitive function was assessed by two validated cognitive tests: the Symbol Digit Modalities Test (SDMT) and the Montreal Cognitive Assessment (MoCA) (Nasreddine et al., 2005; Pascoe et al., 2018). The tests were performed in a quiet room with only the participant and examiner present. The SDMT was administered in oral form to reduce the influence of motor impairments (e.g., tremor). It assesses processing speed, attention, and working memory (Ryan et al., 2020). The number of correct responses in 90 s was selected for further analysis. The MoCA assesses short-term memory, visuospatial abilities, executive functions, attention, concentration, working memory, and language (Nasreddine et al., 2005). A cut point of ≤ 25 was used as an indication of mild cognitive impairment (MCI) (54); however, individuals were included in the study if they could understand simple instructions and were able to participate in the required tests.

### Statistical analysis

Variables modeled as continuous data were assumed to follow a normal distribution and mean ± standard deviation (SD) was calculated unless stated otherwise. Linear regression analyses were performed between the dependent (iron concentration in the SNpc, FWF in the PSN, whole- and regional brain volumes, and the SDMT and MoCA scores) and independent outcomes (VO_2_max and P_max_). Associations adjusted for age and sex were calculated using multiple linear regression analyses. Model validation was performed by inspecting the standard residuals (plots of the standardized residuals against fitted, QQ-plots, and plots of the standardized residuals against each variable). Correlations ≥ 0.90 were considered very strong (corresponding to r2 ≥ 0.81), 0.70–0.89 as strong (corresponding to r2 between 0.49 and 0.79), 0.40–0.69 as moderate (corresponding to r2 between 0.16 and 0.48), 0.10–0.39 as weak (corresponding to r2 between 0.01 and 0.15), and < 0.10 as negligible (corresponding to r2 < 0.01) (Schober et al., 2018). Statistical analyses were performed using Stata 17.0 (StataCorp LLC). Statistical significance was set at *p* ≤ 0.05.

## Results

**Fig. 1:**
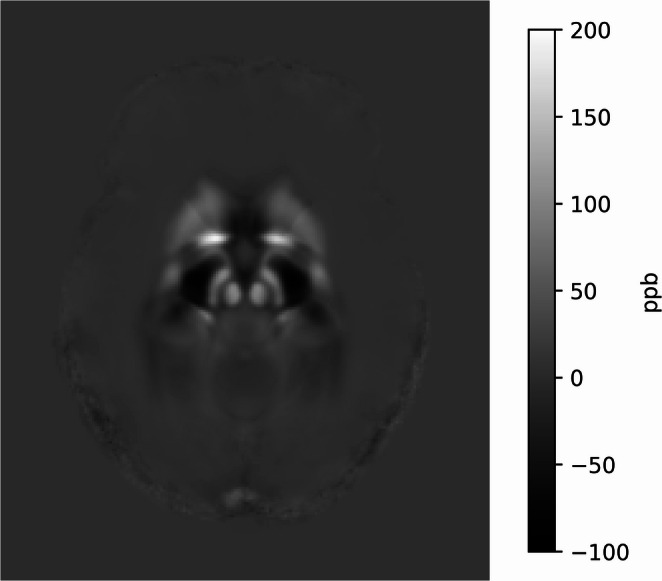
Average pixel intensity of the substantia nigra on QSM across all participants. ppb = parts per billion

A total of 105 participants were included between April 2020 and December 2022. MRI scans from two participants were discarded due to invalid images. Additionally, whole-brain volume data for two participants, QSM images for eight participants, and FWF data for seven participants were discarded because of poor image quality. Figure 1 illustrates the average pixel intensity across all participants within the substantia nigra on QSM. Moreover, six VO_2_max tests were excluded due to incomplete test data. The demographics of the study population are presented in Table 1.

**Table 1:** Demographic, physical, cognitive, and clinical measures of the participants

Variable	n	Mean (± SD)	Range
Sex (m/w)*	105	58/47	
Age (years)	105	60.1 (10.0)	(40–80)
Height (cm)	105	175 (9)	(156–196)
Weight (kg)	105	77.3 (15.3)	(44.1–119.1)
Body mass index (kg/m^2^)	105	25.1 (3.9)	(15.4–34.1)
Time since diagnosis (median (IQR)) (years)	105	2 (1;4)	(0.25–5)
Hoehn & Yahr score (median (IQR)) (1–5)	105	2 (2;2)	(1–3)
Levodopa equivalent daily dose (mg)	105	400 (212)	(80–1548)
VO_2_max (ml O_2_/kg/min)	99	28.9 (7.8)	(13.6–49.2)
Peak power (W/kg)	101	15.2 (4.3)	(4.5–26.9)
MoCA (score)	105	27.3 (2.4)	(18–30)
SDMT (score)	105	49.4 (12.3)	(12—76)
**MDS-UPDRS**			
I (0–52)	102	8.9 (5.5)	(0–31)
II (0–52)	102	6.7 (5.3)	(0–28)
III (0–132)	105	20.6 (11.3)	(1–60)
IV (median (IQR)) (0–24)	105	1 (0;4)	(0–12)

Abbreviations: *VO2max* Cardiorespiratory fitness, *Pmax* Peak muscle power, *ppb* parts per billion, *MoCA* Montreal Cognitive assessment, *SDMT* Symbol Digit Modalities Test, *MDS-UPDRS* Movement Disorder Society-Sponsored Revision of the Unified Parkinson’s Disease Rating Scale, *IQR* Interquartile range. *Number of men and women

### MRI and cognitive outcomes

Simple linear regression analyses (Table 2) revealed several associations between VO_2_max and brain volumes. Except for hippocampal volume and total gray matter volume, P_max_ was associated with all brain volume outcomes. All associations were weak (*r*^2^ = 0.06–0.14). Furthermore, VO_2_max and P_max_ showed weak to moderate associations with both cognitive function measures (*r*^2^ = 0.12–0.21).

**Table 2:** Simple (model 1) and multivariate (model 2) regression analyses including VO_2_max and peak power as independent variables and MRI outcomes and cognitive function as dependent variables

Dependent variable	Independent variable(s)	Coefficient [95%CI]	Standardized beta coefficient	r^2^
SNpc iron concentration	1. VO_2_max 2. VO_2_max Age Sex^#^	−0.39 [−1.83;1.04] −0.35 [−2.13;1.42] −0.47 [−0.89;1.82] −22.64 [−45.78;0.51]	−0.06 −0.05 0.09 −0.21	0.00 0.05
	1. Peak power 2. Peak power Age **Sex**^#^	−1.31 [−3.38;0.76] −0.90 [−3.16;1.36] −0.43 [−0.73;1.58] **−21.77 [−42.83;−0.70]**	−0.12 −0.09 0.08 −0.20	0.02 0.06
PSN free water fraction	1. VO_2_max 2. VO_2_max **Age** **Sex**^**#**^	−0.001 [−0.003;0.001] −0.000 [−0.002;0.002] **0.002** [**0.00;0.003**] **−0.042** [**−0.068;−0.016**]	−0.12 −0.02 **0.28** **−0.32**	0.01 0.20
	1. Peak power 2. Peak power Age **Sex**^#^	−0.002 [−0.005;0.001] −0.001 [−0.004;0.002] 0.001 [−0.000;0.003] **−0.033** [**−0.174;0.412**]	−0.16 −0.07 0.20 **−0.24**	0.02 0.13
Whole-brain volume (mL)	**1. VO**_**2**_**max** 2. VO_2_max **Age** **Sex**^**#**^	**1.63** [**0.68;2.59**] 0.51[−0.50;1.52] **−1.93** [**−2.70;−1.16**] **19.55** [**6.42;32.69**]	**0.33** 0.10 **−0.50** **0.25**	**0.11** 0.39
	**1. Peak power** 2. Peak power **Age** **Sex**^#^	**2.69** [**1.28;4.10**] 0.95 [−0.34;2.24] **−2.03** [**−2.68;−1.37**] **17.19** [**5.20;29.18**]	**0.35** 0.12 **−0.52** **0.22**	**0.12** 0.42
Hippocampus volume (mL)	1. VO_2_max 2. VO_2_max Age **Sex**^**#**^	0.00 [−0.02;0.01] 0.00 [−0.01;0.02] 0.00 [−0.01;0.02] **0.25** [**0.02;0.49**]	−0.03 0.07 0.09 **0.23**	0.00 0.01
	1. Peak power 2. Peak power Age **Sex**^#^	0.00 [−0.02;0.02] 0.00 [−0.02;0.03] 0.00 [−0.01;0.02] **0.24** [**0.02;0.45**]	0.11 0.04 0.08 **0.21**	0.00 0.05
Putamen volume (mL)	**1. VO**_**2**_**max** 2. VO_2_max **Age** **Sex**^**#**^	**0.02** [**0.00;0.04**] 0.01 [−0.02;0.03] **−0.03** [**−0.05;−0.01**] **0.34** [**0.00;0.69**]	**0.20** 0.06 **−0.33** **0.20**	**0.04** 0.18
	**1. Peak power** 2. Peak power **Age** Sex^#^	**0.05** [**0.01;0.08**] 0.03 [−0.01;0.06] **−0.03** [**−0.04;−0.01**] 0.27 [−0.06;0.59]	**0.27** 0.15 **−0.29** 0.15	**0.08** 0.17
Caudate volume (mL)	**1. VO**_**2**_**max** 2. VO_2_max **Age** Sex^#^	**0.03** [**0.00;0.05**] 0.01 [−0.02;0.04] **−0.03** [**−0.06;−0.01**] 0.34 [−0.07;0.74]	**0.20** 0.05 **−0.33** 0.16	**0.04** 0.16
	**1. Peak power** 2. Peak power **Age** Sex^#^	**0.06** [**0.02;0.10**] 0.04 [−0.34;0.01] **−0.03** [**−0.05;−0.01**] 0.29 [−0.08;0.67]	**0.30** 0.19 **−0.26** 0.14	**0.09** 0.08
Pallidus volume (mL)	**1. VO**_**2**_**max** 2. VO_2_max **Age** **Sex**^**#**^	**0.01** [**0.01;0.02**] 0.01 [−0.00;0.02] **−0.02** [**−0.02;−0.01**] **0.19** [**0.05;0.32**]	**0.32** 0.14 **−0.41** **0.25**	**0.10** 0.32
	**1. Peak power** **2. Peak power** **Age** **Sex**^**#**^	**0.03** [**0.01;0.04**] **0.01** [**0.00;0.03**] **−0.02** [**−0.02;−0.01**] **0.18** [**0.06;0.30**]	**0.37** **0.18** **−0.44** **0.24**	**0.14** **0.37**
Thalamus volume (mL)	1. VO_2_max 2. VO_2_max **Age** **Sex**^**#**^	0.02 [−0.01;0.06] 0.00 [−0.04;0.04] **−0.05** [-**0.08;−0.02**] **0.96** [**0.43;1.49**]	0.12 0.00 **−0.35** **0.34**	0.02 0.25
	**1. Peak power** 2. Peak power **Age** **Sex**^**#**^	**0.07** [**0.02;0.12**] 0.04 [−0.01;0.09] **−0.04** [**−0.06;−0.01**] **0.95** [**0.48;1.43**]	**0.27** 0.15 **−0.27** **0.34**	**0.07** 0.26
Total gray matter volume (mL)	**1. VO**_**2**_**max** 2. VO_2_max **Age** **Sex**^**#**^	**1.40** [**0.31;2.49**] 0.16 [−0.01;0.00] **−2.09** [**−2.99;−1.18**] **19.71** [**3.73;34.69**]	**0.25** 0.03 **−0.49** **0.22**	**0.06** 0.31
	1. Peak power 2. Peak power **Age** **Sex**^**#**^	1.15 [−0.49;2.80] −0.86 [−0.34;0.01] **−2.36** [**−3.14;−1.57**] **15.70** [**1.38;30.03**]	0.14 −0.10 **−0.55** **0.18**	0.02 0.08
Total white matter volume (mL)	1. VO_2_max 2. VO_2_max Age Sex^#^	0.23 [−0.06;1.10] 0.35 [−0.74;1.45] 0.16 [−0.68;1.00] 0.35 [−13.95;14.64]	0.06 0.08 0.05 0.01	0.00 0.00
	**1. Peak power** **2. Peak power** Age Sex^#^	**1.54** [**0.30;2.77**] **1.81** [**0.44;3.18**] 0.33 [−0.37;1.03] 1.48 [−11.31;14.27]	**0.24** **0.28** 0.10 0.02	**0.06** **0.07**
MoCA	**1. VO**_**2**_**max** **2. VO**_**2**_**max** Age Sex^#^	**0.10** [**0.04;0.15**] **0.09** [**0.02;0.16**] −0.01 [−0.07; 0.04] 0.26 [−0.62;1.14]	**0.34** **0.32** −0.06 0.06	**0.12** **0.12**
	**1. Peak power** **2. Peak power** Age Sex^#^	**0.16** [**0.08;0.25**] **0.13 [0.03;0.22]** −0.04 [−0.09;0.01] −0.04 [−0.92;0.84]	**0.35** **0.27** −0.18 −0.01	**0.12** **0.14**
SDMT	**1. VO**_**2**_**max** **2. VO**_**2**_**max** **Age** **Sex**^**#**^	**0.65** [**0.37;0.92**] **0.42** [**0.13;0.71**] **−0.46** [**−0.68;−0.23**] **7.46** [**3.63;11.29**]	**0.43** **0.28** **−0.39** **0.31**	**0.18** **0.43**
	**1. Peak power** **2. Peak power** **Age** **Sex**^**#**^	**1.10** [**0.69;1.52**] **0.64** [**0.25;1.03**] **−0.53** [**−0.73;−0.34**] **5.34** [**1.72;8.96**]	**0.46** **0.27** **−0.44** **0.22**	**0.21** **0.44**

Abbreviations: *VO*_*2*_*max* Cardiorespiratory fitness (ml O_2_/kg/min), *Peak power* W/kg, *SNpc* substantia nigra pars compacta, *PSN* posterior substantia nigra, *MoCA* Montreal Cognitive assessment, *SDMT* Symbol Digit Modalities Test. * = men served as reference group. In bold indicates significant association (*p* < 0.05)

After adjusting for age and sex, associations between VO_2_max and cognition (SDMT, MoCA), as well as between P_max_ and total white matter volume, pallidus volume, and cognition (SDMT), remained significant.

## Discussion

The present study found that, in simple regression analyses, objective measures of physical fitness (i.e., VO_2_max, P_max_) were associated with several brain volumetric outcomes, including whole-brain volume, and cognitive function (i.e., MoCA, SDMT). However, when adjusted for age and sex, only associations between physical fitness and cognition as well as between P_max_ and two brain volumetric outcomes (i.e., total white matter volume, pallidus volume) remained. These findings support a link between two commonly used physical outcomes reflecting different body systems (i.e., cardiovascular and neuromuscular) and cognitive function as well as brain volume in PD. Hypothetically, this suggests that pwPD may positively impact both brain structure and function by increasing VO_2_max and P_max_ through increased exercise participation. However, future well-designed studies (i.e., large, long-term RCTs with well-defined populations) are needed to confirm the causality of these associations.

### VO_2_max and markers of neurodegeneration

The simple analyses showed positive associations between VO_2_max and brain volumetric outcomes as well as cognitive function. However, adjustment for confounding factors is essential when measuring VO_2_max and P_max,_ as both age and sex are known to substantially impact these variables. Subsequently, only the association with cognitive function remained when adjusting for age and sex. These results align with existing cross-sectional data from several studies that show a positive association between VO_2_max and cognitive function in older adults Barnes et al., 2003, Colcombe & Kramer, 2003, Farrell et al., 2018, Sofi et al., 2010.

Although a causal relationship between VO_2_max and cognitive function cannot be inferred from the present study, the association between these two could indicate a protective effect of exercise on cognitive function in pwPD. However, the current literature investigating exercise-induced protection against cognitive decline is somewhat contradictory. A meta-analysis summarizing data from 2049 participants (both healthy and clinical populations) reported exercise to induce significant improvements in attention, processing speed, executive function, and memory (Smith et al., 2010). Similarly, two reviews from 2014 and 2018 concluded that there is a growing body of evidence suggesting significant positive effects of exercise on global cognitive function and a reduced risk of cognitive impairment in pwPD (Murray et al., 2014; da Silva et al., 2018). However, the reported effects were small to moderate, and the reviews did not include meta-analyses. Murray and colleagues concluded that AE could improve cognitive function, although the optimal type, amount, and duration of exercise were still unclear (Murray et al., 2014). Of note, two meta-analyses from 2014 and 2015 found no evidence from RCTs that AE had any benefits on cognitive function in healthy adults (Kelly et al., 2014; Young et al., 2015). However, evidence from animal studies shows exercise-induced protection from cognitive and motor deficits as well as an increased expression of long-term potentiation in the SN, indicating an effect of exercise on the restoration of striatal synaptic plasticity (Marino et al., 2023). Taken together, future well-designed longitudinal studies investigating the effects of exercise on cognition in pwPD are warranted.

This is the first study to investigate the cross-sectional link between VO_2_max and SN iron content, making a direct comparison to previous findings impossible. One hypothesis entails that AE protects dopaminergic neurons from degeneration through enhanced mitochondrial function (Chistiakov et al., 2014; Cotman et al., 2007; Hirsch & Hunot, 2009; Radak et al., 2008). However, no significant associations between VO_2_max and iron content in SN were found in the present study. This may be explained by the fact that iron content was not markedly increased in the present PD population. Providing some support for this hypothesis, a previous study by An et al. (2018) reported a higher mean magnetic susceptibility among pwPD when compared to the levels observed in the present study (i.e., 179.6 ± 65.7 vs 149.9 ± 54.4) (An et al., 2018). These differences in SN magnetic susceptibility may partly be explained by differences in age and disease severity among the participants. However, another study from 2021 found increased SN iron content among pwPD in all disease stages compared to healthy controls (Li et al., 2022).

The present study found no associations between VO_2_max and brain volumetric outcomes when adjusting for age and sex. In contrast, several previous cross-sectional and review studies have reported positive associations between VO_2_max and brain volume in non-PD cohorts (Zhu et al., 2015). Furthermore, an intervention study reported an increase in hippocampal volume after a 1-year AE intervention in healthy elderly adults (Erickson et al., 2011). However, these results have not been replicated in subsequent studies (Erickson et al., 2009; Jonasson et al., 2017; Langeskov-Christensen et al., 2021). Recent meta-analyses investigating the effects of AE interventions on brain volumetric outcomes have found conflicting evidence (Gogniat et al., 2021; Hvid et al., 2021; Johansson et al., 2020). One of the reasons for the discrepancy between cross-sectional and longitudinal findings could be that a cross-sectional VO_2_max assessment reflects the physical activity level of the person`s lifestyle over recent years, whereas RCT interventions usually last a few months. In this context, it is somewhat surprising that the present study found no association between VO_2_max and MRI measures after adjusting for sex and age.

### P_max_ and markers of neurodegeneration

Associations were initially observed between P_max_ and most MRI outcomes as well as cognitive function. However, after adjusting for age and sex only the associations with pallidus volume, total white matter volume, and cognitive function remained. No studies have previously investigated the association between P_max_ and brain volume in PD. Yet, muscle strength was negatively associated with atrophy of the hippocampus in Alzheimer’s Disease patients (Moon et al., 2019). This suggests that RE may positively impact brain volumetric outcomes in populations with neurological diseases, but such putative effects need causal confirmation in future PD RCTs. In support of the neuroprotective effect of RE, an RCT found reduced white matter atrophy in 155 older healthy women following 52 weeks of RE (Best et al., 2015). Additionally, increased hippocampal volume has been reported in older women undertaking 24 weeks of RE (Kim et al., 2017). Animal studies suggest that these effects transpire through exercise-induced increases in brain-derived neurotrophic factor (BDNF) levels (Pinho et al., 2019), insulin-like Growth factor-1 (Cassilhas et al., 2012), and expression of antioxidant molecules, mediated by upregulation of nuclear factor erythroid 2–related factor 2 (Ma, 2013; Pinho et al., 2019; Souza et al., 2017).

The evidence for RE-induced protection against cognitive decline in PD is likewise limited. The present study found a positive association between P_max_ and cognitive function, within the domains of working memory, executive function, and visuospatial function. These associations have not been investigated previously in PD. However, several studies have investigated whether RE can either improve cognition or reduce the risk of cognitive decline in non-PD cohorts. Some studies found significant protective effects of RE on cognitive function, whereas others did not (Best et al., 2015; Heyn et al., 2004; Kelly et al., 2014; Landrigan et al., 2020; Li et al., 2018). Best et al. reported improved executive function and memory in a cohort of elderly women following 52 weeks of RE (Best et al., 2015). These results are supported by several reviews and meta-analyses that reported an effect of RE on different cognitive outcomes (Heyn et al., 2004; Kelly et al., 2014; Landrigan et al., 2020; Li et al., 2018). However, the effects were generally small to moderate, and the included studies reported heterogeneous results (Landrigan et al., 2020), highlighting the need for future RCTs to establish whether RE impacts cognitive function in PD. In this regard, intervention studies should incorporate interventions lasting longer than six months, as markers of brain structure (and function) likely adapt more slowly than parameters related to physical function and maximal muscle strength and power.

### Future research and clinical application

The present study adds further support to the potential neuroprotective effects of physical exercise in pwPD and can inform future RCTs evaluating such effects when selecting outcomes. Future research should focus on establishing whether long-term AE and RE interventions, respectively, induce protective effects that slow cognitive decline and accumulation of SN iron content. Such studies should preferably last years instead of months, as such a timeframe might be necessary to induce significant and measurable neuroprotective effects in pwPD. Also, comprehensive cognitive assessments should be used instead of brief screening tools. Furthermore, future cross-sectional studies investigating associations between physical fitness and markers of neurodegeneration should also be carried out in more advanced PD. Lastly, future studies should consider investigating potential synergistic effects (positive or negative) of combined training consisting of both RE and AE, compared to single-modality exercise interventions.

### Limitations

The present study has several limitations. First, the cross-sectional study design makes it impossible to conclude on causal neuroprotective effects of VO_2_max, P_max_, or physical exercise in PD (Wang And Cheng, 2020). The findings of this study, however, can be used to generate hypotheses about the role of VO_2_max and P_max_ on markers of neurodegeneration in PD. Second, a potential recruitment bias exists in the present study. It is possible that the participants of the present study had a higher physical activity level compared with the average level in pwPD, as this phenomenon has been reported in other physical activity studies (Harris et al., 2008). Third, the MoCA and SDMT are brief screening tools rather than comprehensive cognitive assessments. The MoCA, though widely used, provides only a brief global overview and may lack sensitivity to subtle or domain-specific cognitive impairments in PD (Faust-Socher et al., 2019). The SDMT is a well-validated and widely used measure of processing speed in multiple sclerosis, but its application in PD is more limited. However, in this study, the SDMT was administered orally to minimize motor confounds, and while visual deficits could theoretically influence performance, evidence indicates that lower scores in PD primarily reflect cognitive rather than visuomotor impairment (Pascoe et al., 2018). The present findings should be interpreted with these methodological limitations in mind. Lastly, the presence of tremors among the pwPD could be the cause of several discarded MRI scans, resulting in fewer valid MRI-derived results.

## Conclusions

The present study found that physical fitness, measured as VO_2_max and lower extremity P_max_, showed a weak to moderate association with markers of brain function (i.e., MoCA, SDMT). Additionally, P_max_ showed a moderate association with brain structure (i.e., total white matter and pallidus volume). These exploratory findings further support a potential neuroprotective effect of exercise in pwPD and may help future RCTs in selecting outcomes to evaluate the neuroprotective effects of exercise.

## Data Availability

Data Availability: The data supporting the findings of this study are not publicly available due to restrictions imposed by the General Data Protection Regulation (GDPR). Access to the data is therefore limited to protect the privacy and confidentiality of the participants.

## Abbreviations

PD: Parkinson’s Disease
AE: Aerobic exercise
RE: Resistance exercise
(MDS-)UPDRS: (Movement Disorder Society-Sponsored Revision of) the Unified Parkinson’s Disease Rating Scale
VO_2_max: Maximal oxygen consumption
P_max_: Peak muscle power
pwPD: People with Parkinson’s Disease
MRI: Magnetic resonance imaging
QSM: Quantitative susceptibility mapping
WB: Whole brain
GM: Gray matter
WM: White matter
SN: Substantia nigra
SDMT: Symbol Digit Modalities Test
MoCA: Montreal Cognitive Assessment
